# Retraction Note: Autophagy-mediated degradation of NOTCH1 intracellular domain controls the epithelial to mesenchymal transition and cancer metastasis

**DOI:** 10.1186/s13578-026-01544-9

**Published:** 2026-02-19

**Authors:** Sahib Zada, Jin Seok Hwang, Trang Huyen Lai, Trang Minh Pham, Mahmoud Ahmed, Omar Elashkar, Wanil Kim, Deok Ryong Kim

**Affiliations:** 1 Department of Biochemistry and Convergence Medical Science, Institute of Health Sciences, Gyeongsang National University College of Medicine, Jinju, Republic of Korea; 2 Present Address: Cancer Biology and Immunology Laboratory, College of Dental Medicine, Columbia University Irving Medical Center, New York, NY USA

**Retraction Note to: Cell & Bioscience (2022) 12:17**

10.1186/s13578-022-00752-3

The Editor-in-Chief has retracted this article because of concerns regarding a number of figures presented in this work and the authenticity of the statistical data reported in Figures 2 and 3. These concerns call into question the article's overall scientific soundness. An investigation conducted after its publication discovered the following issues:

- Bands in Figures 1A and 2A appear to overlap with bands in Figures 1A, 1C, and 3A in [1];
- The HeLa/ULK1 and HeLa/NOTCH1 bands in Figure 2A appear to overlap with each other;
- The 0h/CRISPR/Cas9-Atg7 and 0h/CRISPR/Cas9-Atg7+starved assays in Figure 6 appear to overlap with each other;
- There are similarities between the standard deviations of different statistical groups reported in Figure 2B and Figures 3H and 3I.

The authors were unable to provide original source data on request from the Publisher. The Editor-in-Chief therefore no longer has confidence in the integrity of the research presented in this article.

The authors have not stated clearly whether they agree or disagree with this retraction.
